# Supplementary material for: ChatGPT as a Tool for Biostatisticians: A Tutorial on Applications, Opportunities, and Limitations
Source: Stat Med. 2025 Oct 23;44(23-24):e70263. doi: 10.1002/sim.70263 (PMC12548020; doi:10.1002/sim.70263)
Supplement: Supplementary file 1 — Data S1. Supporting Information. [file SIM-44-0-s001.zip › Supplementary Materials Additional.zip!/3.1_Systematic_Reviews_and_Meta_aspects/3.1_Forest Plot - 4o - Chat 10.pdf]

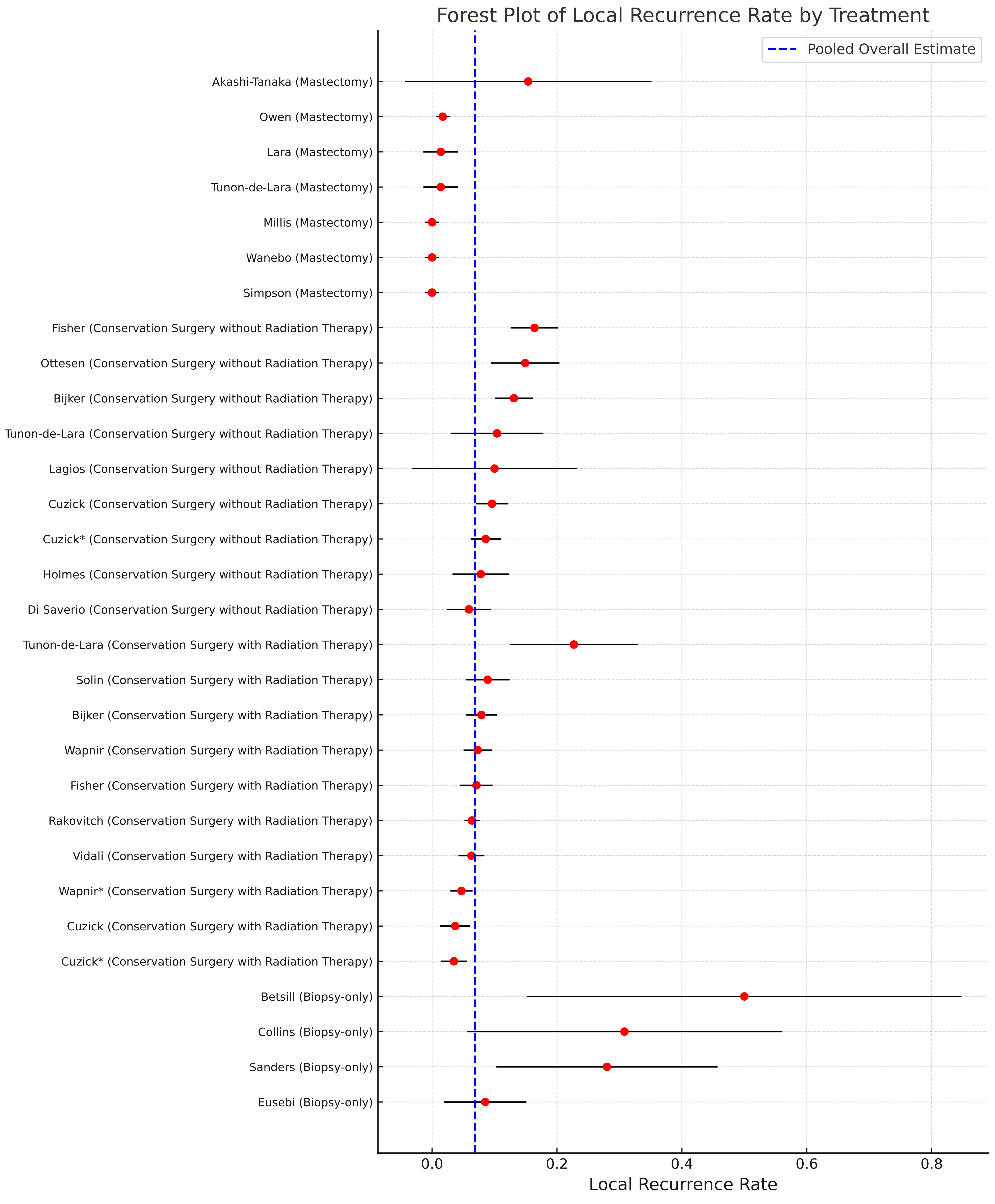
Forest Plot of Local Recurrence Rate by Treatment
Pooled Overall Estimate
Akashi-Tanaka (Mastectomy)
Owen (Mastectomy)
Lara (Mastectomy)
Tunon-de-Lara (Mastectomy)
Millis (Mastectomy)
Wanebo (Mastectomy)
Simpson (Mastectomy)
Fisher (Conservation Surgery without Radiation Therapy)
Ottesen (Conservation Surgery without Radiation Therapy)
Bijker (Conservation Surgery without Radiation Therapy)
Tunon-de-Lara (Conservation Surgery without Radiation Therapy)
Lagios (Conservation Surgery without Radiation Therapy)
Cuzick (Conservation Surgery without Radiation Therapy)
Cuzick* (Conservation Surgery without Radiation Therapy)
Holmes (Conservation Surgery without Radiation Therapy)
Di Saverio (Conservation Surgery without Radiation Therapy)
Tunon-de-Lara (Conservation Surgery with Radiation Therapy)
Solin (Conservation Surgery with Radiation Therapy)
Bijker (Conservation Surgery with Radiation Therapy)
Wapnir (Conservation Surgery with Radiation Therapy)
Fisher (Conservation Surgery with Radiation Therapy)
Rakovitch (Conservation Surgery with Radiation Therapy)
Vidali (Conservation Surgery with Radiation Therapy)
Wapnir* (Conservation Surgery with Radiation Therapy)
Cuzick (Conservation Surgery with Radiation Therapy)
Cuzick* (Conservation Surgery with Radiation Therapy)
Betsill (Biopsy-only)
Collins (Biopsy-only)
Sanders (Biopsy-only)
Eusebi (Biopsy-only)
0.0
0.2
0.4
0.6
0.8
Local Recurrence Rate
